# Polyglycolic Acid Shield Prevents Repeated Delayed Bleeding After Endoscopic Stricturotomy for Anastomotic Stricture in Crohn's Disease: A Case Report

**DOI:** 10.1002/deo2.70420

**Published:** 2026-08-27

**Authors:** Hidezumi Kikuchi, Taka Asari, Tetsuya Tatsuta, Kazuhiro Nagane, Kentaro Hoshi, Shohei Igarashi, Takato Maeda, Yasuhisa Murai, Shinji Ota, Hirotake Sakuraba

**Affiliations:** ^1^ Division of Endoscopy Hirosaki University Hospital Aomori Japan; ^2^ Department of Gastroenterology Hematology, and Clinical Immunology Hirosaki University Graduate School of Medicine Aomori Japan

**Keywords:** complication, Crohn's disease, endoscopic strictuotomy, hemostasis, polyglycolic acid

## Abstract

Endoscopic stricturotomy (ES) is a novel approach for treating benign strictures of the lower gastrointestinal tract. However, it is performed at a limited number of facilities, and strategies for preventing complications have not yet been established. We performed ES for ileo‐sigmoid anastomosis associated with Crohn's disease (CD). Although endoscopic dilatation was successful, delayed bleeding occurred. Endoscopic hemostasis provided temporary relief, but delayed bleeding recurred nine times within 17 days after ES. Combined hemostatic treatments with vascular forceps soft coagulation, biocompatible synthetic peptide gel, and endoscopic clips failed to stop recurrent bleeding. Infliximab infusion was also unsuccessful. Finally, wound coverage with polyglycolic acid (PGA) sheets prevented rebleeding and avoided abdominal surgery. Here, we report that covering with a PGA sheet and fibrin glue may be useful in preventing recurrent delayed bleeding after ES for anastomosis associated with CD.

## 1 Introduction

Lower gastrointestinal strictures include malignant and benign types caused by colorectal cancer and inflammation, respectively. While surgery and metallic stent placement are effective for malignant strictures, the treatment of benign strictures depends on the patient's condition and the medical level of the facilities.

Traditionally, endoscopic balloon dilation (EBD) has been widely used to treat benign strictures, including those associated with Crohn's disease (CD) [1]. Except for the cases of strongly angulated strictures with adhesions, EBD is generally considered a safe technique [2]. However, a single EBD procedure may not be sufficient for prolonged and adequate dilatation, leading to repeated treatments.

Recently, endoscopic stricturotomy (ES) has been reported as an endoscopic treatment for benign intestinal strictures [3, 4, 5]. The procedure involves an incision of the narrowed lesion of the intestinal tract with an electrosurgical knife and relief of the stricture. Literature on lower gastrointestinal ES is limited, and strategies for preventing complications have not yet been established.

We performed ES for the ileo‐sigmoid anastomotic stricture associated with CD. Although dilatation with ES was successful, delayed bleeding occurred nine times within 17 days after ES. Here, we report that covering with a polyglycolic acid (PGA) sheet and fibrin glue (FG) is effective in preventing recurrent delayed bleeding after ES for anastomotic strictures in CD.

## 2 Case

A 52‐year‐old man was hospitalized for abdominal pain. He had experienced recurrent colitis since his 18‐year‐old and was diagnosed with ulcerative colitis at a local hospital. At the age of 29 years, he visited our department because of resistance to medical treatment and required a colectomy. During the operation, the surgeon observed ileitis with fat wrapping and altered the surgical approach to subtotal colectomy with ileo‐sigmoid anastomosis, suspecting CD. Postoperative pathological examination, which revealed non‐caseating granulomata and ileal longitudinal ulcers, suggested that the patient had CD. Therefore, he was treated with an injection of infliximab (10 mg/kg, every 8 weeks) and oral administration of 6‐mercaptopurine (50 mg/day). He did not receive any antithrombotic drugs. In his 33‐year‐old, a stricture at the postoperative anastomosis progressed, and the colonoscope could not pass through. The patient received repeated EBD and enteral nutrition as needed.

**FIGURE 1:**
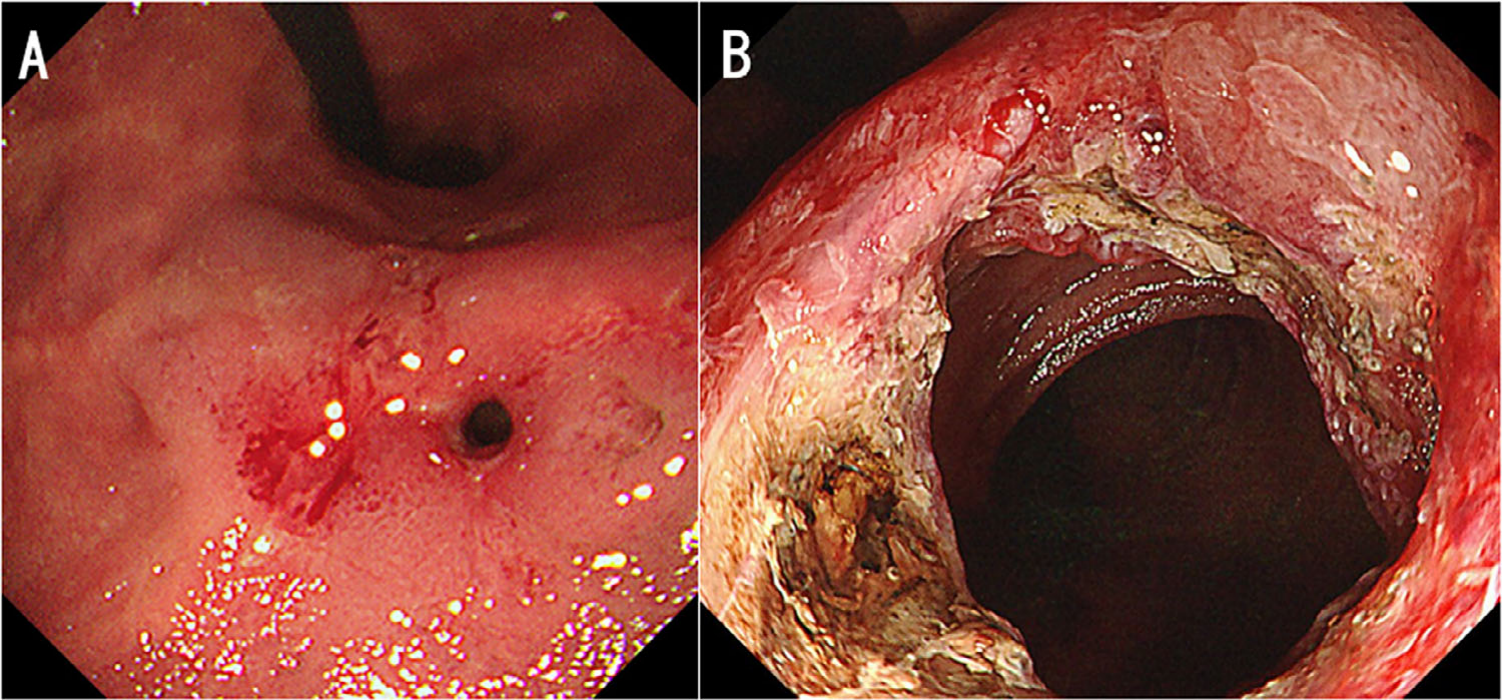
(A) Ileo‐sigmoid anastomosis with stricture in a patient with Crohn's disease. (B) Endoscopic image immediately after successful endoscopic stricturotomy.

At his admission at 52 years, blood tests showed inflammatory biomarkers within the normal range: white blood cells 4220/µL, albumin 4.3 g/dL, C‐reactive protein 0.09 mg/dL, leucine‐rich alpha‐2 glycoprotein 7.0 µg/mL. Colonoscopy revealed that the anastomotic stricture had worsened (Figure 1A). His abdominal pain temporarily improved after fasting and enteral nutrition therapy, and he was discharged after dietary guidance. Two months later, the patient was readmitted for ES of the anastomotic stricture. ES was performed using an electrosurgical knife (IT‐nano; Olympus, Tokyo, Japan). The ES procedure was initiated with several vertical incisions followed by a circumferential incision in the stricture. Dilatation was insufficient even after complete exposure of the muscle layer. Therefore, the stricture was additionally expanded up to 16 mm via EBD, which resulted in significant dilatation (Figure 1B).

**FIGURE 2:**
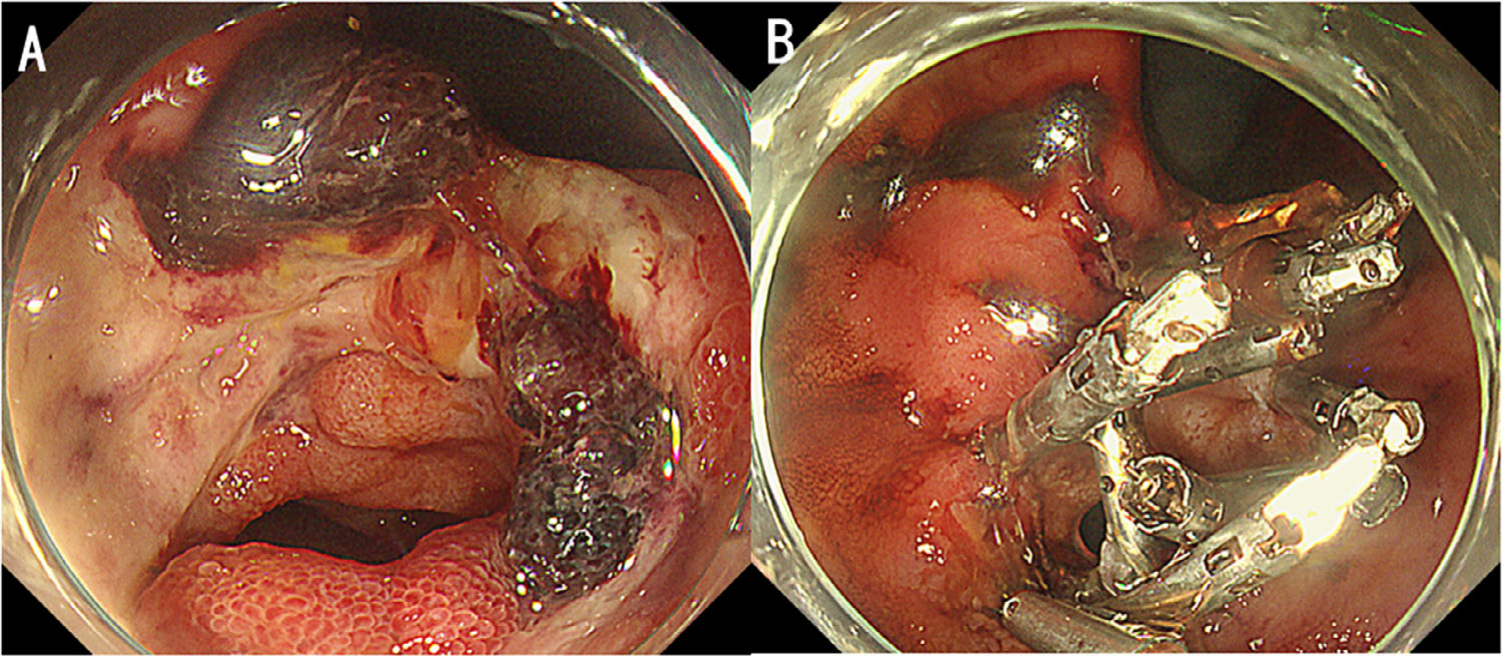
Nine days after endoscopic stricturotomy, delayed bleeding occurred, and an endoscopic examination revealed a blood clot (A). The wound was closed using Mantis clips (B).

**FIGURE 3:**
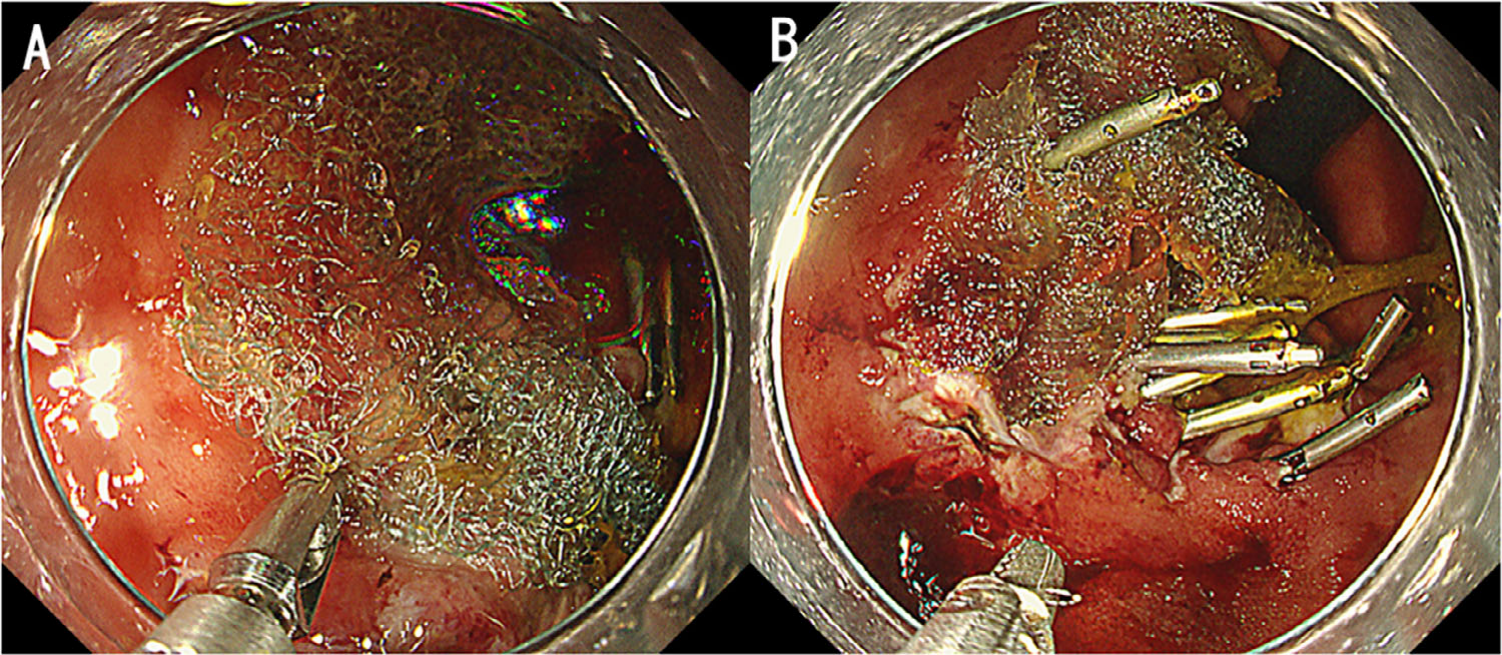
(A, B) Wound coverage with a polyglycolic acid sheet for an open ulcer after clip detachment.

**FIGURE 4:**
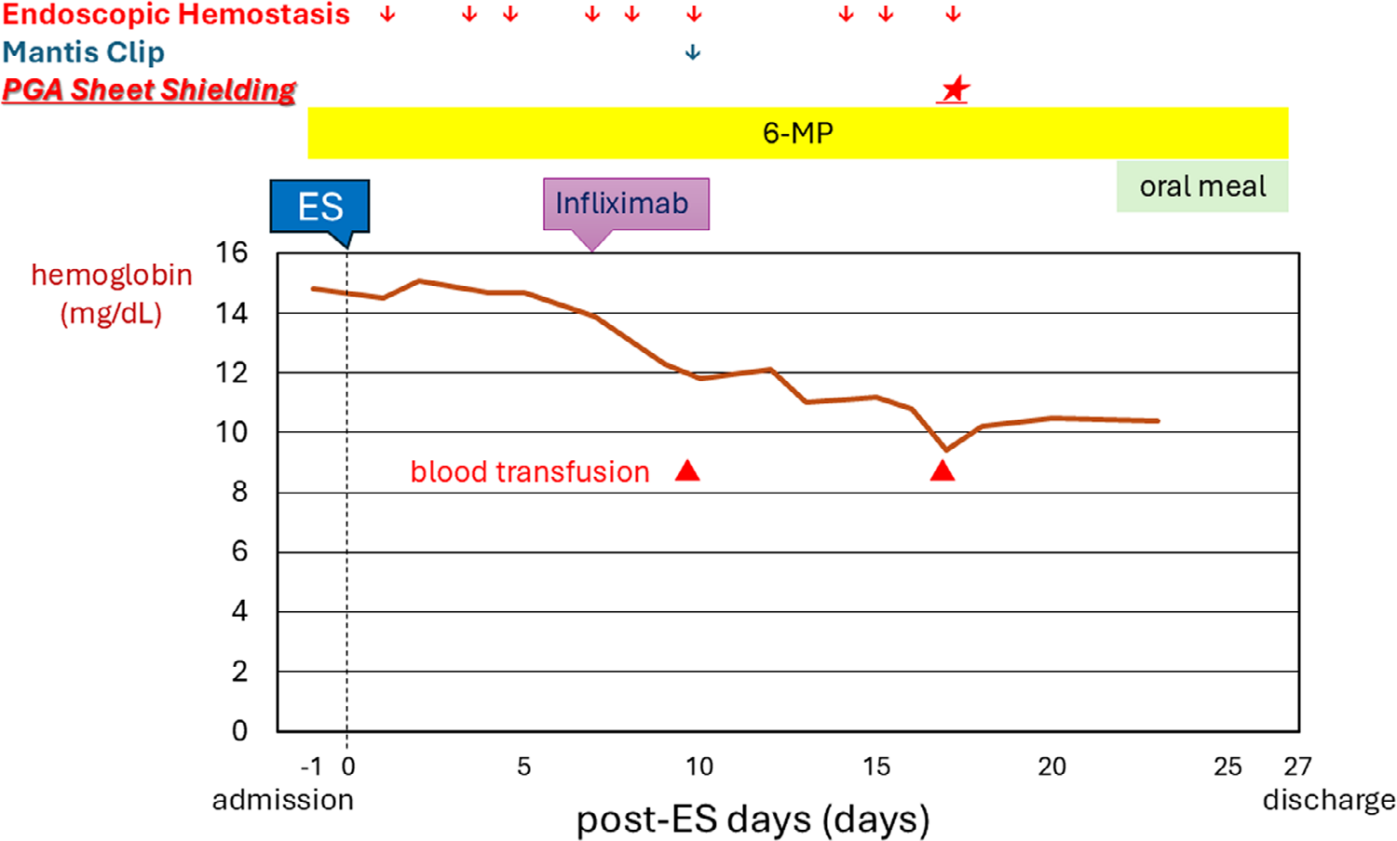
Clinical Course. 6‐MP, 6‐mercaptopurine; ES, endoscopic stricturotomy.

However, the patient experienced nine episodes of delayed bleeding on days 1, 3, 4, 6, 7, 9, 14, 15, and 17 after ES. At each bleeding time, endoscopic hemostasis was performed using hemostatic forceps (Coagraspar; Olympus, Tokyo, Japan) and a biocompatible synthetic peptide gel (PuraStat; 3D Matrix, Tokyo, Japan). These hemostatic procedures temporarily stopped bleeding but did not completely prevent recurrent delayed bleeding. Since his bleeding episodes recurred five times in the first week after ES, he was administered infliximab as cyclic treatment on post‐ES day 7, but it was ineffective. On post‐ES days 9 and 17, his blood pressure significantly dropped, and blood transfusions were performed. On post‐ES day 9, the ES ulcer was closed with large endoscopic clips (MANTIS Closure Device; Boston Scientific Japan, Tokyo) (Figure 2). However, some clips detached, causing further bleeding from the open ulceration on post‐ES day 14. On post‐ES day 17, the patient complained of bloody stools and subsequently developed hypotension. Therefore, after endoscopic hemostasis using hemostatic forceps, the ulcer was covered with a PGA sheet (NEOVEIL; GUNZE MEDICAL, Tokyo, Japan) and an FG (BOLHEAL; Meiji, Tokyo, Japan) (Figure 3). Finally, no further bleeding was observed, and the patient resumed eating on post‐ES day 22. The patient was discharged on post‐ES day 27 (Figure 4).

## 3 Discussion

This is the first report describing the successful management of repeated delayed bleeding after lower gastrointestinal ES using PGA sheets and FG. ES is an effective approach for lower gastrointestinal strictures; however, it is performed in a limited number of facilities. Although ES can provide greater dilation than EBD, its indications and strategies for preventing complications are still not clearly established. In our case, recurrent delayed bleeding occurred after ES for anastomotic strictures in a patient with CD, and the wound covered with PGA sheets and FG was effective in preventing rebleeding.

Regarding complications associated with ES, it has been reported that delayed bleeding occurred 18.5% in ES for benign stenosis of the lower gastrointestinal tract; all cases were from CD patients [6]. Furthermore, in the ES for anastomotic stricture associated with CD, blood transfusion was required in 8.2% due to delayed bleeding [7]. The reason for the high incidence of post‐ES bleeding in anastomotic strictures of CD is unknown, but the presumable theories are as follows: 1) anastomotic ulcers tend to develop easily after CD surgery, thus delaying ulcer healing; 2) abundant blood vessels are newly generated at the anastomosis due to prolonged inflammation and hypoxia in CD; and 3) upper intestinal fluid with pancreatic juice directly stimulates ulcers at the ileo‐colonic anastomosis and promotes bleeding. On the other hand, the possibility remains that the additional EBD influenced the delayed bleeding, while it contributed to sufficient dilation. Therefore, endoscopists should be aware that the risk of post‐ES bleeding might increase with additional EBD.

In our case, vascular coagulation with hemostatic forceps and administration of infliximab were insufficient to prevent rebleeding. However, tight wound closure with large MANTIS clips contributed to a longer interval until the next bleeding episode. Unfortunately, clip detachment resulted in rebleeding, suggesting that wound closure may be effective in preventing rebleeding. If the fresh wound after ES had been closed with clips earlier, the risk of dropping off might have been reduced, which could have been effective in preventing post‐ES bleeding.

Tissue shielding with PGA sheets is a common technique to prevent delayed bleeding after gastric endoscopic submucosal dissection (ESD) [8, 9]. Additionally, the efficacy of PGA sheets has been established in the prevention of esophageal strictures after ESD [10]. Therefore, we considered that covering the wound with a PGA sheet was a favorable approach to prevent rebleeding in this case. This suggested that cauterization of the responsible bleeding vessel might be insufficient in recurrent delayed bleeding after ES in patients with CD and that covering the wound was crucial. Furthermore, coating post‐ES ulcers may reduce intestinal fluid exposure and prevent delayed bleeding. Based on our experience, PGA shielding might be a suitable approach when several days or more have elapsed, while clipping is considered effective for preventing delayed bleeding in the early period post‐ES.

This study has several limitations. First, it stands for only a single case, and therefore, the efficacy of PGA shielding cannot be generalized. Additionally, because the PGA sheet was introduced on post‐ES day 17, it is possible that the natural healing process influenced the results.

In conclusion, we argue that wound coverage with PGA sheets and FG may be effective in preventing and managing recurrent delayed bleeding after ES for anastomotic strictures in patients with CD.

## Author Contributions

**Hidezumi Kikuchi**: conception and manuscript writing. **Taka Asari**, **Tetsuya Tatsuta**, **Kazuhiro Nagane**, **Shohei Igarashi**, and **Kentaro Hoshi**: endoscopy specialists. **Takato Maeda**, **Yasuhisa Murai**, and **Shinji Ota**: data collection. **Hirotake Sakuraba**: revision of the article for intellectual content.

## Funding

The authors have nothing to report.

## Ethics Statement

The patient was treated within the standard scope of care provided by the National Health Insurance of Japan.

## Consent

Informed consent was obtained from the patient for the medical treatment, and the patient provided informed consent for this publication.

## Conflicts of Interest

The authors declare no conflicts of interest.

## Data Availability

Data sharing is not applicable to this article, as no datasets were generated or analysed during the current study.
